# The Reconstruction of St. Bartholomew's Hospital

**Published:** 1907-07-27

**Authors:** 


### HOSPITAL ADMINISTRATION.

### CONSTRUCTION AND ECONOMICS.

### THE RECONSTRUCTION OF ST. BARTHOLOMEW'S HOSPITAL.

OPENING OF NEW OUT-PATIENT DEPARTMENT BY THE PRINCE OF WALES.

#### The Oldest English Hospital.

On Tuesday last H.R.H. the Prince of Wales opened the new out-patient department at St. Bartholomew's Hospital. The inauguration of the new premises is likely to remain a landmark not only in the history of the hospital itself, but equally in that of hospital management in the metropolis generally. The oldest of our hospitals, the institution founded by Rahere, patronised by Henry I. and Edward VI., and enlarged and added to by Henry VIII., has shown itself sufficiently youthful and enthusiastic to become one of the pioneers of scientific hospital out-patient reform in London. Other hospitals have improved and enlarged their out-patient departments, some of them, as the Evelina Hospital for Sick Children, with a conspicuous measure of success, but none have attempted the task of reorganisation and reconstruction so systematically or upon such sound scientific principles as St. Bartholomew's. The extensions, which were opened on Tuesday last, mark an advance which to those acquainted only with the cramped, unwholesome surroundings that obtained in all out-patient departments in the past, and are still unfortunately present in many of our charitable institutions, will seem as astonishing as it is admirable.

A thorough inspection of the new buildings has convinced us that they fail in very few particulars, if they fail at all, to substantiate the claim that they will add greatly to the efficiency of the hospital or to justify the hope that they will afford the most perfect methods of observation and treatment for out-patients. The accommodation which they supply is more than ample for existing requirements, and likely to be sufficient for the future—at least for many years. On the ground floor the large central waiting-room for "casualty patients," into which open more than a dozen dressing and examination rooms, and which is in direct communication, by means of separate passages, with dispensary, wards, isolation rooms, and the various special departments, accommodates close upon a thousand patients. On the first floor are the medical and surgical out-patient rooms, each set containing demonstrators', clerks', patients' dressing-rooms, an operating or emergency room, and examining and consulting rooms. On the floors above are housed, equally spaciously, the laryngological, orthopædic, *x*-ray, gynæcological, aural, dental, and ophthalmic departments. In the dispensary block, which is closely connected on basement and ground floor with the main building and at the back with the older part of the hospital, is located the dispensary, which has attached to it, on the ground floor, a spacious waiting-hall capable of seating 250 out-patients. Above this are the kitchen—a model of what such an establishment should be—and the sculleries: below, in the basement, is the well-equipped pharmaceutical laboratory with its cool and airy store-rooms and its mixing-room. In the basement of the main block are the isolation rooms for infectious cases.

Where so much that is excellent and worthy cordial praise is brought under the observer's notice, it is a hard task to single out any feature for special mention. Taking the new buildings as a whole, we think they can honestly be deemed an attempt to secure the maximum of scientific efficiency and thoroughness. To the layman, possibly also to the practitioner who has only in mind the squalid discomfort of old out-patient rooms, the new buildings may seem in many ways a costly and extravagant display of unnecessary lavishness. But a moment's thought will convince one of the necessity for all this attention to detail, this thoroughness that at first sight seems reckless disregard of cost. Shoddiness is not economy, and so far as we have been able to judge, durability and efficiency rather than cheapness are the features of the new department. This is well seen in the remarkably excellent fitting up of the dispensary. Seven windows, including an emergency window, open from the dispensary on to the large waiting-room, allowing thus the serving by the officers of seven different files of patients at the same time—a distinct improvement upon the single-window arrangement that still prevails at most institutions. The dispensary floor is of acid proof asphalt, a costly material, but one which is practically undamagable by caustic or stains, and which in the long run should more than repay its cost. All the rooms from the large central waiting-room to the smallest of the orthopædic dressing-rooms, are tiled in white and parti-coloured tiles, which not only adds to their appearance, but from a sanitary point of view is almost a necessity in such a building. The corners are rounded, and the fittings are everywhere of the latest pattern for hospital use, admitting the minimum of dust as deposit, and offering the maximum of comfort and ease in cleaning.

We have been particularly struck with the arrangements for the comfort of the patients as well as of the examining officers. Nothing is of greater importance in such a building than a proper scheme of ventilation and effective drainage. The former has been secured by a modification of the plenum system and by large wide-angle windows which open flush with the frames. The drainage has been specially seen to; all the pipes are exposed to view, and overflows and surplus holes have been reduced to the absolutely necessary limit. There are two large electric lifts in the building, and a wide central staircase and two outside emergency stairways, put each floor into communication with the central waiting-room. In the ophthalmic department several novel features are found: one of these is the excellent dark room for ophthalmoscopic work which permits half a score of patients being simultaneously examined; another the isolated "septic room" for the treatment of cases suffering from gonorrhœal ophthalmia. In the *x*-ray and orthopædic departments a large exercise room, very airy and well lit, and an ingeniously red-tiled developing-room, demand special mention. A feature of each floor is the ample lavatory accommodation which is found on each landing. Another feature, and one which we believe is unique in out-patient departments in this country, are the reserved "surgical" emergency beds on one of the higher floors. Any special case seen in the hall below may be taken up to these and kept under observation before final admittance into the general wards or transference to outside institutions. Such an arrangement cannot but be of the greatest convenience to out-patient officers.

St. Bartholomew's is to be cordially congratulated on its up-to-date out-patient department, and there can hardly be any doubt that the possession of these great conveniences and aids to efficient examination and management will put the hospital in the front of metropolitan institutions, so far at least as out-patient work is concerned. The new department is a model of its kind, embodying the best results which experience and careful thought have combined to produce. The cost of the whole—£130,000 approximately—may seem unduly large to some, but when it is remembered that no good purpose can be served by a cheapness which in the end must mean a false economy, no one can honestly cavil at the expenditure which has produced so excellent a result. From a medico-surgical point of view, the buildings are almost ideal, and work in them will be watched with interest by those who have devoted some attention to reforms in hospital out-patient work.

